# Localization of Cdc7 Protein Kinase During DNA Replication in *Saccharomyces cerevisiae*

**DOI:** 10.1534/g3.117.300223

**Published:** 2017-09-18

**Authors:** Daniel Rossbach, D. Suzi Bryan, Jay R. Hesselberth, Robert Sclafani

**Affiliations:** Department of Biochemistry and Molecular Genetics, University of Colorado Anschutz Medical Campus, Aurora, Colorado 80045

**Keywords:** replication, kinase, calling cards, chromatin, origins

## Abstract

DDK, a conserved serine-threonine protein kinase composed of a regulatory subunit, Dbf4, and a catalytic subunit, Cdc7, is essential for DNA replication initiation during S phase of the cell cycle through MCM2-7 helicase phosphorylation. The biological significance of DDK is well characterized, but the full mechanism of how DDK associates with substrates remains unclear. Cdc7 is bound to chromatin in the *Saccharomyces cerevisiae* genome throughout the cell cycle, but there is little empirical evidence as to specific Cdc7 binding locations. Using biochemical and genetic techniques, this study investigated the specific localization of Cdc7 on chromatin. The Calling Cards method, using Ty5 retrotransposons as a marker for DNA–protein binding, suggests Cdc7 kinase is preferentially bound to genomic DNA known to replicate early in S phase, including centromeres and origins of replication. We also discovered Cdc7 binding throughout the genome, which may be necessary to initiate other cellular processes, including meiotic recombination and translesion synthesis. A kinase dead Cdc7 point mutation increases the Ty5 retrotransposon integration efficiency and a 55-amino acid C-terminal truncation of Cdc7, unable to bind Dbf4, reduces Cdc7 binding suggesting a requirement for Dbf4 to stabilize Cdc7 on chromatin during S phase. Chromatin immunoprecipitation demonstrates that Cdc7 binding near specific origins changes during S phase. Our results suggest a model where Cdc7 is loosely bound to chromatin during G_1_. At the G_1_/S transition, Cdc7 binding to chromatin is increased and stabilized, preferentially at sites that may become origins, in order to carry out a variety of cellular processes.

DNA replication in eukaryotes is a tightly regulated process that ensures the genome is duplicated once and only once during the cell cycle. Failure to control replication mechanisms leads to chromosome instability, aneuploidy, and mutations, all hallmarks of many human diseases (Rossbach and Sclafani 2016). DNA replication is initiated at specific segments of genomic DNA, termed origins of replication, during S phase of the cell cycle. In budding yeast, *Saccharomyces* *cerevisiae*, origins are known as ARSs (autonomous replicating sequences) and contain an 11 bp consensus sequence termed the ACS (ARS consensus sequence) that serves as a docking site for replisome proteins (Sclafani and Holzen 2007). Origins are classified by two key characteristics: efficiency and timing. Origin efficiency is determined by how frequently the origin initiates replication and whether it is used in a population of cells during every cellular division. Origin timing is a temporal reflection of when a given origin initiates replication during S phase. Origins can initiate replication throughout S phase and the time they initiate is quantified by the *T_rep_* (timing of replication), the time it takes to duplicate 50% of the DNA (Raghuraman *et al.* 2001; Yabuki *et al.* 2002).

Initiation of DNA replication is executed in distinct steps: origin licensing and origin firing. During origin licensing, ARSs are bound throughout the cell cycle by the ATP-dependent ORC (origin recognition complex) (Bell and Dutta 2002). The Pre-RC (prerecognition complex) is formed when the MCM complex (minichromosome maintenance helicase), composed of six paralogous proteins Mcm2-7, is loaded onto double-stranded DNA in G_1_ phase of the cell cycle by Cdt1 and Cdc6 (Labib 2010). The MCM helicase loads as an inactive double hexamer during the licensing step of DNA replication (Forsburg 2004; Frigola *et al.* 2013). The origin firing step of DNA replication begins when the MCM helicase is activated in S phase by the sequential action of two kinases, CDK (cyclin-dependent kinase) and DDK (Dbf4-dependent kinase), forming the Pre-IC (preinitiation complex) (Johnston *et al.* 1999; Nougarede *et al.* 2000; Heller *et al.* 2011). The concerted action of the two kinases leads to recruitment of Cdc45, the GINS complex, as well as the polymerases to form the active replisome. Once the replisome is formed, the MCM helicase unwinds DNA and uses polymerases to replicate DNA.

DDK is a serine-threonine protein kinase, highly conserved in eukaryotes from yeast to humans (Sato *et al.* 1997; Hess *et al.* 1998) and is composed of a regulatory subunit, Dbf4, and a catalytic subunit, Cdc7 (Hartwell 1971; Patterson *et al.* 1986; Hollingsworth and Sclafani 1990). Each subunit of DDK contains conserved motifs and domains necessary for binding the other subunit and holding the complex together (Hughes *et al.* 2012). In addition to essential kinase domains conserved in all eukaryotes, budding yeast Cdc7 has a unique 55 amino acid C-terminal domain required for binding Dbf4 (Jackson *et al.* 1993). This C-terminal domain is not found in homologous Cdc7 proteins indicating there are alternate binding mechanisms required to bind Cdc7 to Dbf4 in other eukaryotes (Jackson *et al.* 1993).

Cdc7 and Dbf4 protein subunits are regulated differently during the cell cycle. The Cdc7 protein is stably expressed and subsequently bound to chromatin throughout the entire cell cycle (Weinreich and Stillman 1999; Ferreira *et al.* 2000). Conversely, Dbf4 protein expression oscillates throughout the cell cycle due to transcriptional regulation and protein stability regulation by the APC (anaphase-promoting complex) (Cheng *et al.* 1999; Oshiro *et al.* 1999; Weinreich and Stillman 1999; Ferreira *et al.* 2000). When APC-dependent degradation of Dbf4 ceases upon the cells’ transition from G_1_ to S phase, Dbf4 protein is stable and associates with Cdc7 and chromatin immediately. At the completion of S phase, Dbf4 protein is rapidly degraded again by the APC. Mutations in essential APC subunits or a Dbf4 N-terminal region resembling destruction boxes eliminates APC-mediated degradation (Cheng *et al.* 1999; Ferreira *et al.* 2000). DDK activity is regulated by expression of the Dbf4 subunit in much the same way CDKs are regulated by cyclin activity. In G_1_ phase, when Dbf4 protein is not expressed, catalytic activity of Cdc7 is low. As Dbf4 expression increases at the G_1_/S transition, the catalytic activity of Cdc7 increases and remains high throughout S phase, before decreasing when Dbf4 is degraded at the end of S phase (Cheng *et al.* 1999; Oshiro *et al.* 1999; Weinreich and Stillman 1999; Ferreira *et al.* 2000).

The primary functional role of DDK is to initiate DNA replication through the phosphorylation of the MCM helicase, primarily on subunits Mcm2, Mcm4, and Mcm6 (Lei *et al.* 1997; Sheu and Stillman 2010). DDK phosphorylation of the MCM helicase is proposed to induce a conformational change, and together with CDK activity, permits recruitment of Cdc45, the GINS complex, and DNA polymerases thereby forming an activated replisome (Labib 2010). The requirement for DDK to activate the helicase can be bypassed by expression of an Mcm5 mutant, *mcm5-bob1*, that potentially modifies the structure of Mcm5 and mimics the phosphorylation-induced conformational change (Hardy *et al.* 1997). In addition to the *mcm5-bob1* mutant, deletion or phosphomimetic alterations of DDK phosphorylation sites in the N-terminus of Mcm4 can also bypass Cdc7 loss (Randell *et al.* 2010; Sheu and Stillman 2010). Recent experiments tethering DDK to origins with Gal4 results in overall increases in origin efficiency, while FRAP shows DDK diffuses rapidly in the nucleus such that it randomly interacts with origins during S phase to initiate productive phosphorylation events (Patel *et al.* 2008). While DDK’s most well characterized role is in DNA replication, it also functions to regulate other chromatin-bound substrates in meiotic recombination, translesion synthesis (TLS) during DNA-damage-induced mutagenesis, chromosome cohesion, and centrosome duplication (Sasanuma *et al.* 2008; Natsume *et al.* 2013; Brandao *et al.* 2014). Therefore, Cdc7’s functional role in the cell is that of a master regulator to promote recruitment of other proteins that carry out these processes.

Dbf4 protein has been shown by one hybrid screen (Dowell *et al.* 1994) and chromatin immunoprecipitation (ChIP) in G_1_-arrested cells (Katou *et al.* 2006; Natsume *et al.* 2013) to directly associate with origins of replication. While this has provided useful information for the Dbf4 regulatory subunit, similar methods have not been able to successfully identify where the Cdc7 subunit binds within the genome. The inability of conventional methods to associate endogenous Cdc7 with origins of replication is likely due to the transient interaction of the kinase with substrates. In this study, we adapt established methods and modify conventional ones to understand binding of the Cdc7 subunit within the genome.The Calling Cards method was developed to map binding sites of transcription factors within the budding yeast genome (Wang *et al.* 2008). This method takes advantage of the Ty5 retrotransposon in budding yeast where Ty5 mRNA is reverse transcribed into double-stranded cDNA and translocated to the nucleus where the Ty5 integrase reintegrates Ty5 retrotransposons into the genome near heterochromatin and telomeric regions under the direction of the Sir4 heterochromatin protein. Calling Cards fuses transcription factors to the domain of the Sir4 heterochromatin protein that interacts with the Ty5 integrase. Therefore, Ty5 retrotransposon cDNA is integrated into the genome near to where a given transcription factor binds, leaving a permanent mark of its association with that region of the genome (Xie *et al.* 2001). Furthermore, barcodes can be added to transcription factor fusion constructs to allow for analysis of multiple transcription factors in a single study (Wang *et al.* 2007, 2011). While initial studies used this method primarily for transcription factor binding sites, its design permits modification for analyzing binding sites of any DNA binding protein including replication factors.

Conventional methods have been unable to precisely determine Cdc7 protein binding sites at specific genomic locations, so we adapted Calling Cards for replication proteins to look at genome wide Cdc7 binding by means of where Ty5 retrotransposon permanent integrations are made. An additional benefit of Calling Cards is its ability to analyze protein binding sites in dividing cells over multiple generations, where previous studies have been limited to arrested populations. Calling Cards fusion constructs were generated fusing the domain of Sir4 that interacts with Ty5 integrase to DNA replication factors Cdc7 and Sld3. These fusion constructs were expressed in budding yeast containing a Ty5 donor plasmid under control of the GAL1 promoter as well as a deletion of the Sir4 protein. Growth of these strains on media plates containing galactose induces transcription of Ty5 mRNA from the donor plasmid that is then reversed transcribed into cDNA. The donor Ty5 plasmid contains a *HIS3* marker permitting selection of cells with genomically integrated Ty5 retrotransposons. Genomic DNA can be isolated, fragmented by restriction digestion and circularized by intramolecular ligation thereby recovering the DNA immediately flanking a Ty5 retrotransposon integration. The DNA flanking Ty5 retrotransposon integrations is amplified using inverse PCR, subjected to Illumina Sequencing, and mapped back to the budding yeast genome. This provides qualitative information as to where replication proteins bind chromatin as well as a quantitative measurement of how many Ty5 retrotransposons integrations are made.

## Materials and Methods

### Yeast strains, plasmids, and media

Yeast strains and plasmids are listed in Table 1 and Table 2, respectively. *CDC7* (wild type) open reading frame was amplified for Calling Cards from pRS277 (Table 2) using primers A and B (Table 3) (Hollingsworth *et al.* 1992). *cdc7*(*N168A*) open reading frame was made by PCR mutagenesis of pCH766 (Table 2) with primers J and K (Table 3) and amplified for Calling Cards by primers A and B. *cdc7*(*N168A*)(*1–452*) was amplified for Calling Cards from pCH777 (Table 2) with primers A and C (Table 3). Calling Cards plasmids containing DNA replication factors fused to the Ty5 integrase interacting domain of Sir4 were made using previously published methods (Wang *et al.* 2008). Fusion construct homologous recombination was done in yeast strain RSY452 (Table 1) and and Sanger sequencing with primers L and M (Table 3). Fusion constructs were verified for functionality by protein expression and complementation of *cdc7* temperature sensitive mutant in RSY302 (Table 1). Verified fusion constructs were transformed into yeast strain RSY1354 (Table 1) containing the Ty5 donor plasmid and selected on Glucose–Ura–Trp media plates. Yeast strains were grown according to standard procedures and Calling Cards selection was carried out according to previous methods (Wang *et al.* 2008). For ChIP strains, pCH766 and pCH777 plasmids (Table 2) were transformed into yeast strain RSY1294 (Table 1) and selected on –His media plates.

**Table 1:** *S. cerevisiae* strains used

Yeast Strain	Genotype	Background/Reference
RSY302	*MAT* α *leu2* *trp1* *his3Δ1* *ura3* *cdc7-7*	A364a/Jackson *et al.* (1993)
RSY452	*MAT*a *ura3* *trp1* *his3*Δ*1* *leu2* *can1* *cyh2*	A364a
RSY1294	*MAT*a *ade2-1* *his3-11* *15* *leu2-3* *112* *trp1-1* *ura3-1* *can1-100* *bar1*::*hisG*	W303
RSY1352	YM7635 (pBM5249) (pRAS746)	S288C
RSY1354	YM7635 (pBM5249)	S288C
DRY101	YM7635 (pBM5249) (pDR001)	S288C
DRY106	RSY1294 (pCH766)	S288C
DRY107	RSY1294 (pCH777)	S288C
DRY110	YM7635 (pBM5249) (pBM4607)	S288C
DRY113	YM7635 (pBM5249) (pDR011)	S288C
DRY126	YM7635 (pBM5249) (pDR016)	S288C
YM7635	*MAT*a*/MATα his3Δ1/his3Δ1 leu2Δ0/leu2Δ0 ura3Δ0/ura3Δ0 met15Δ0/MET15 lys2Δ0/LYS2 sir4*::*Kan/sir4*::*Kan trp1*::*Hyg/trp1*::*Hyg*	S288C/Wang *et al.* (2008)

**Table 2:** Recombinant plasmids used

Plasmid	Genotype	Reference
pBM4607	*ARS CEN TRP1 aldhp-GAL4-SIR4-MYC*	Wang *et al.* (2011)
pBM5249	*ARS CEN URA3 Gal1p-TY5-HIS3 PEBar1*	Wang *et al.* (2008)
pCH766	*2µ HIS3 Gal10pro-4xHA-CDC7*	Hardy and Pautz (1996)
pCH777	*2µ HIS3 Gal10pro-3xHA-cdc7*(*N168A*)	Hardy and Pautz (1996)
pDR001	*ARS CEN TRP1 aldhp-CDC7-SIR4-MYC*	This study
pDR011	*ARS CEN TRP1 aldhp-cdc7*(*N168A*)*-SIR4-MYC*	This study
pDR016	*ARS CEN TRP1 aldhp-cdc7*(*N168A*) (*1–452*)*-SIR4-MYC*	This study
pRAS746	*ARS CEN TRP1 aldhp-SLD3-SIR4-MYC*	This study
pRS277	*ARS CEN LEU2 CDC7*	Hollingsworth *et al.* (1992)

**Table 3:** Primer sequences

Primer	Sequence
(A) Cdc7-Sir4 Fusion Forward	ATACAATCAACTCCAAGCTTGAAGCAAGCCTCCTGAAAGATGACAAGCAAAACGAAGAA
(B) Cdc7-Sir4 Fusion Reverse	TTTGGGTTTGCTAGAATTAGTATCACTATGCGACACTCTTTCAGATATTAGGAGAACATCCTT
(C) Cdc7-Sir4 C-terminal deletion Fusion Reverse	TTTGGGTTTGCTAGAATTAGTATCACTATGCGACACTCTTTCGAAGCATTGTTCCAAAACCTG
(D) Inverse PCR Forward Primer (OM8714)	AATGATACGGCGACCACCGAGATCTACACTCTTTCCCTACACGACGCTCTTCCGATCTAATTCACTACGTCAACA
(E) Inverse PCR Illumina TruSeq Index Primer 1 (OM8827)	CAAGCAGAAGACGGCATACGAGATCGTGATCGGTCTCGGCATTCCTGCTGAACCGCTCTT
(F) Inverse PCR Illumina TruSeq Index Primer 2	CAAGCAGAAGACGGCATACGAGATACATCGCGGTCTCGGCATTCCTGCTGAACCGCTCTT
(G) Inverse PCR Illumina TruSeq Index Primer 3	CAAGCAGAAGACGGCATACGAGATGCCTAACGGTCTCGGCATTCCTGCTGAACCGCTCTT
(H) Inverse PCR Illumina TruSeq Index Primer 4	CAAGCAGAAGACGGCATACGAGATTGGTCACGGTCTCGGCATTCCTGCTGAACCGCTCTT
(I) PE Read 2 Illumina Sequencing Primer	GATCGGAAGAGCGGTTCAGCAGGAATGCCGAGACCG
(J) Cdc7 N168A Forward	CATCAAACCGACAGCTTTTTTATTTAATTTGGAATTGGG
(K) Cdc7 N168A Reverse	CAAATTAAATAAAAAAGCTGTCGGTTTGATGTCTCTATG
(L) Fusion Verification Forward Primer (OM6189)	CACAATATTTCAAGCTATACC
(M) Fusion Verification Reverse Primer (OM6373)	CTCATCAACCAACGAAACGG
(N) ARS306 Forward	TGCGAATTTCCTGTTCAGTG
(O) ARS306 Reverse	TATTGGGATTGGGGGCTAAT
(P) ARS607 Forward	GGCTCGTGCATTAAGCTTGT
(Q) ARS607 Reverse	GCAAATCAAAGGATCCCTCA
(R) ARS305 + 8 kb Forward	GGGTCTATGCCCTCGTGATA
(S) ARS305 + 8 kb Reverse	ATCATACCTCGCCAATCTCG
(T) ARS1 + 10 kb Forward	TGCTTTCAAGCTGGTCTTCA
(U) ARS1 + 10 kb Reverse	TTTCTCTGCGCAGTTTTTCA

### Calling Cards efficiency assay

Calling Cards strains were grown in Glucose–Ura–Trp media and then plated on Galactose–Ura–Trp plates to induce Ty5 transcription into mRNA. Cells were replicated to YPD plates and removed from plates. An Invitrogen Countess was used to count cells for serial dilutions. Serial dilutions were plated on either YPD plates (for total viable cells) or –His FOA plates (selecting for Ty5 transpositions only) and colonies were counted. The ratio of colonies grown on –His FOA to the total number of cells on YPD was calculated to measure the efficiency of Ty5 transposition.

### Calling Cards library preparation

Calling Cards libraries were prepared according to previous methods (Wang *et al.* 2008) with the following modifications. 100 OD_600_ genomic DNA was harvested using the following protocol: cells were resuspended in a solution containing 1 M Sorbitol, 50 mM Tris-HCl, pH = 8.0, and 10 mM 2-Mercaptoethanol (BME). Cells were spheroplasted with 0.7 mg/ml 100 K Zymolyase (US Biological) and shaken at 30° for 1 hr. The resultant spheroplasts were resuspended in a solution containing 1 M Sorbitol, 1% SDS, 20 mM EDTA and incubated at 55° overnight. Phenol:Choloform:Isoamyl alcohol (25:24:1) extraction followed by Chloroform:Isoamyl alcohol (24:1) extraction and RNase treatment (10 mg/ml) was done and the DNA was then precipitated with 3 M Sodium Acetate and 100% Ethanol. One microgram of genomic DNA was digested with *Hin*dIII, *Hpa*II, or *Taq*α1 (New England BioLabs). Digested DNA (200 ng) was used for intramolecular ligation reaction by T4 Ligase (New England BioLabs). Ligated DNA (20 µl) was used for inverse PCR amplification with 0.25 mM dNTPs, 0.125 µM of primer D (Table 3), 0.125 μM of primer E, F, G, or H (Table 3), Taq polymerase, and Taq buffer (35 mM MgCl_2_, 750 mM KCl, 0.1% TritonX-100, pH = 8.7). Inverse PCR amplification was done with 31 cycles of 95° for 30 sec, 60° for 30 sec, and 72° for 3 min. Each PCR reaction was diluted to 10 nM and the three digestion reactions for a given Calling Cards strain were pooled and submitted for sequencing on the Illumina GAII or MiSeq with primer I (Table 3).

### Analysis of Calling Cards sequencing

Sequences were analyzed by alignment to a reference genome (*sacCer1*) using Bowtie (Langmead *et al.* 2009) and SAMtools (Li *et al.* 2009), processed to bedGraph format using BEDTools (Quinlan and Hall 2010), and visualized in the UCSC genome browser (Karolchik *et al.* 2011). Coverage at each position was normalized by the signal at each genomic position relative to the number of aligned reads in the library (*i.e.*, reads per million, RPM) to get quantitative information or normalized to the mean number of unique insertions to get specific positional information. Software and pipeline used to analyze Calling Cards data can be found at https://github.com/hesselberthlab/callingcards.

### ChIP and quantitative PCR

ChIP was performed as previously described (Kogut *et al.* 2009) with the following modifications. Yeast strains were grown in –His 2% Raffinose media overnight. Cells were diluted in YEP + 2% Raffinose + 2% Galactose to induce Cdc7 protein overexpression and grown to 0.8 OD_600_. Cells were synchronized with 50 nM α factor (αF) for 4 hr before being released into media containing 10 mg/ml pronase. Fifty milliliters of cells was harvested before addition of αF to serve as an asynchronous sample, at *T* = 0 when pronase was added to serve as a G_1_-arrested population and at 15-min intervals for 90 min after pronase addition to look at different S phase time points. Immunoprecipitations were set up using 300 µl of chromatin solution and incubated with 25 µg anti-HA antibody (12CA5; Roche) for 15 hr at 4°. Three hundred microliters of chromatin solution was mock treated as no antibody control and 1.5 ml of chromatin solution was used as input. Protein G sepharose beads were incubated with immunoprecipitations (IPs) and no antibody controls for 1 hr at 4°. Beads were washed according to previously described methods (Ramey and Sclafani 2014) and resuspended in 200 µl ChIP elution buffer and 40 µl TE then incubated overnight at 65°. RNase (3 mg/ml) was added to IPs and no antibody controls and incubated for a minimum of 30 min at 37°. Four microliters of 20 mg/ml Proteinase K was added to IPs and controls and incubated for 2 hr at 42°. Organic extraction of IPs and controls using Phenol:Choloform:Isoamyl alcohol (25:24:1) was followed by ethanol precipitation. IP and control pellets were resuspended in 150 µl TE and input pellets were resuspended in 300 µl TE. ChIP chromatin was subjected to quantitative PCR, using 2× sybergreen master mix from (Roche) and a Roche Lightcycler 480 thermocycler. ChIP chromatin was subjected to qPCR analysis with primers N-U (Table 3) (Aparicio *et al.* 1997; Ramey and Sclafani 2014).

### Data availability

Strains and plasmids are available upon request. Supplemental Material, Figure S1 in File S1 contains UCSC genome browser Calling Cards data for Gal4 loci on Chromosomes 2, 4, and 12. Figure S2 in File S1 contains UCSC genome browser Calling Cards data at specific origins of replication. Figure S3 in File S1 contains comparison of Calling Cards insertions events and transcription start sites or centromeres.

Sequencing data generated in this study has been deposited in NCBI GEO under accession GSE103943. https://www.ncbi.nlm.nih.gov/geo/query/acc.cgi?acc=GSE103943.

## Results

### Cdc7 kinase dead fusion constructs efficiently integrate Ty5 transposons into the genome

**Figure 1:**
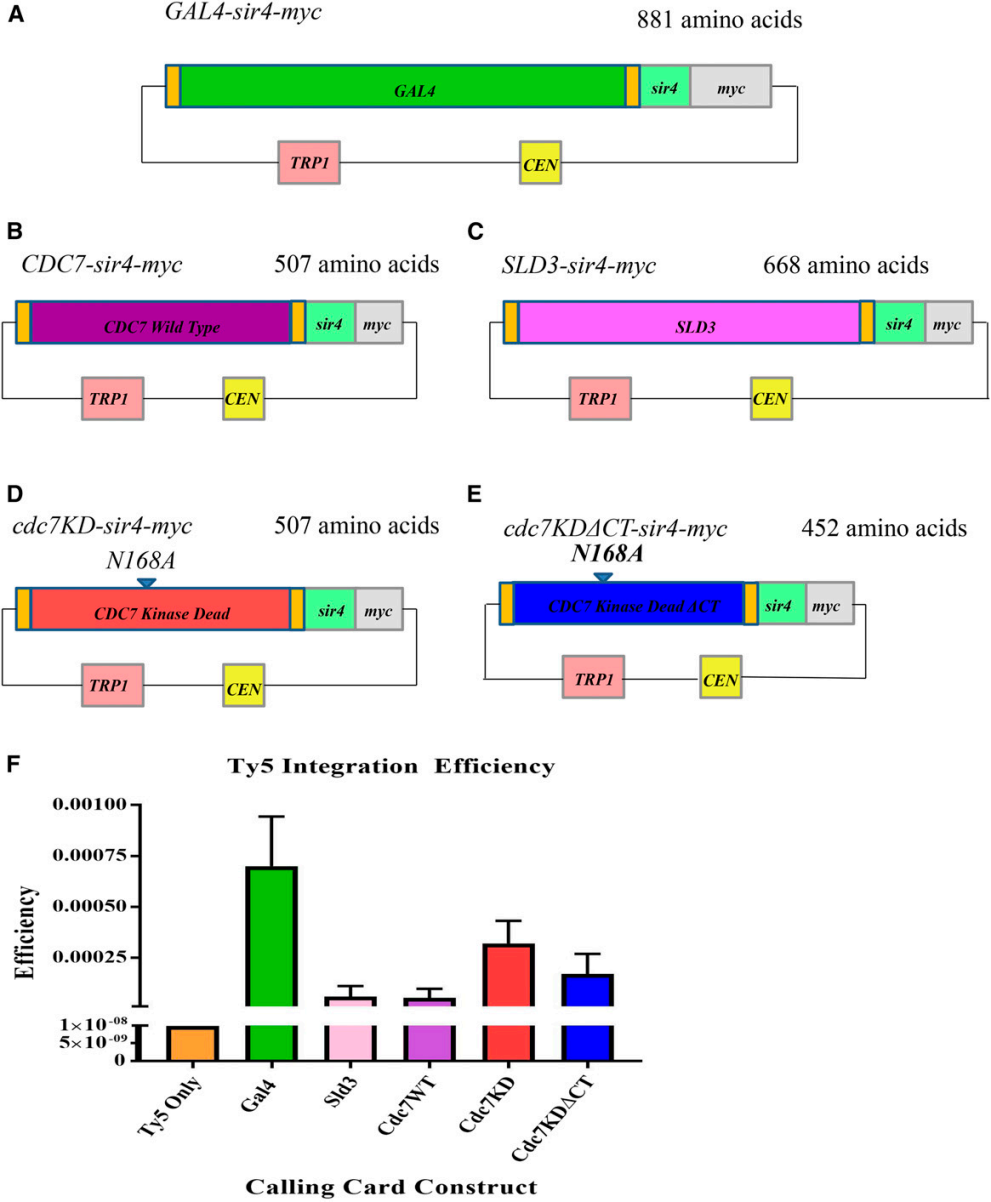
Calling Cards construct efficiency. (A) *GAL4* open reading frame fused to the *sir4* domain that interacts with Ty5 integrase. This construct was donated to us by David Mayhew (Washington University in St. Louis). (B) Full length *CDC7* (507 aa) was inserted into the *GAL4-sir4-myc* plasmid backbone to create *sir4*-fusion. (C) *SLD3* fused to *sir4*. (D) Full length *cdc7KD* (507 aa) fused to *sir4* contains a N168A kinase dead point mutation. (E) *cdc7KDΔCT* fused to *sir4* contains same N168A mutation and additionally lacks 55 C-terminal residues. (F) Yeast strain containing Ty5 transposon donor plasmid was transformed with the indicated DNA replication factor fusion constructs and tested for efficiency to integrate Ty5 transposons into genomic DNA by measuring the relative amount of His+ 5FOA resistant cells, in which transposition occurred.

Calling Cards has been established using fusion constructs as being a sensitive and quantitative method for analyzing genomic DNA binding sites of transcription factors (Wang *et al.* 2007, 2008). In our Calling Cards experiments, the *GAL4-sir4-myc* fusion construct (designed by the Mayhew laboratory at Washington University–St. Louis) was used as a positive control (Figure 1A). This construct was used as a template to make fusion constructs containing wild-type *CDC7* and *SLD3* (Figure 1, B and C). In addition to wild-type *GAL4*, *CDC7*, and *SLD3*, Calling Cards constructs were made using mutant *cdc7* alleles carrying mutations affecting its binding to chromatin. A kinase dead construct with a -bp point mutation in the catalytic domain of Cdc7 changing asparagine (N) to alanine (A) at amino acid 168 (Figure 1D) and a kinase dead construct that also contains a truncation of the final 55 amino acid C-terminal residues were made (Figure 1E). Homologous recombination of the replication factors into the fusion construct was verified by Sanger sequencing and the construct functionality was verified by Western blot analysis using the myc tag in the fusion and complementation of a *cdc7-7* temperature sensitive strain (data not shown).

To validate the efficacy of Calling Cards using DNA replication factor proteins, the efficiency of each fusion construct to integrate Ty5 retrotransposons into the genome was determined (Figure 1F). Integration efficiency of each replication factor fusion construct was compared to the efficiency of a strain containing only the Ty5 donor plasmid without a Sir4 fusion construct as well as to the efficiency of the *GAL4-sir4* transcription factor fusion construct. Strain RSY1354 (Table 1), containing only the *Ty5-HIS3* donor plasmid under the *GAL1* promoter without a Sir4 fusion construct, integrated Ty5 transposons inefficiently into the genome at a rate of 10^−9^/cell. In contrast, strain DRY110 (Table 1), containing the *GAL4-sir4* fusion construct, integrated Ty5 transposons efficiently into the genome at a rate of 5 × 10^−4^/cell, comparable to the previously reported value 10^−5^/cell (Wang *et al.* 2008).

The efficiency of the DNA replication factor fusion constructs is expected to be within the range set by background Ty5 transposon integrations, the negative control, and Gal4-directed Ty5 transposon integrations, the positive control. Strains DRY101 (Table 1), containing the wild-type *CDC7-sir4-myc* fusion construct, and RSY1352, containing the *SLD3-sir4-myc* fusion construct, integrate Ty5 transposons at rates of 4 × 10^−5^/cell and 7 × 10^−5^/cell, respectively. Compared to *GAL4-sir4*, *CDC7-sir4* and *SLD3-sir4* fusions integrate Ty5 transposons less efficiently.

Strain DRY113 (Table 1) contains the kinase dead *cdc7*(*N168A*)*-sir4-myc* fusion construct (*cdc7KD-sir4*) that prevents Cdc7 from phosphorylating MCM helicases (Hardy and Pautz 1996). This construct was used to potentially increase Ty5 transposon integration events compared to the wild-type *CDC7* fusion construct with the rationale that blocking substrate phosphorylation would result in Cdc7 protein kinase remaining bound to the substrate longer. As shown originally in the crystal structure of Protein Kinase A, the conserved asparagine residue is essential for catalytic activity as it interacts directly with the catalytic basic residue (Knighton *et al.* 1991). Thus, the *cdc7*(N168A) protein still binds ATP but is unable to transfer phosphate to the substrate. Increased binding of substrates *in vivo* by protein kinase dead mutants has been shown for both MEK5 kinase (Wu *et al.* 1996) and for Rad53 kinase (Holzen and Sclafani 2010). Ty5 integration efficiency of *cdc7KD-sir4* was determined to be 3 × 10^−4^/cell, sevenfold more than wild-type *CDC7-sir4* and similar to the *GAL4-sir4* efficiency, consistent with increased binding by the Cdc7 kinase dead protein.

Strain DRY123 contains the kinase dead C-terminal truncation Cdc7 fusion construct *cdc7*(*N168A*)(*1–452*)*-sir4-myc* fusion (*cdc7KDΔCT-sir4*), which prevents essential Dbf4 binding to Cdc7. This construct was used to investigate the role of Dbf4 in localizing Cdc7 to chromatin (Jackson *et al.* 1993). *cdc7KDΔCT-sir4* had a Ty5 transposon integration efficiency of 1.69 × 10^−4^/cell, fourfold higher than *CDC7-Sir4*. Our calculated efficiencies suggest *cdc7KD-sir4* and *cdc7KDΔCT-sir4* constructs are substantially better at integrating the Ty5 transposon into the genome compared to *CDC7-sir4* or *SLD3-sir4*. As postulated, the difference in efficiency is likely due to the extended binding of the kinase dead protein to substrates. Given their higher integration efficiencies, *GAL4-sir4*, *cdc7KD-sir4*, and *cdc7KDΔCT-sir4* were used in the Calling Cards experiment to determine Cdc7 binding to chromatin in the budding yeast genome, while *CDC7-sir4* and *SLD3-sir4* will largely be ignored as a result of their low integration efficiency.

### Cdc7 fusion constructs integrate Ty5 transposons throughout the genome

**Figure 2:**
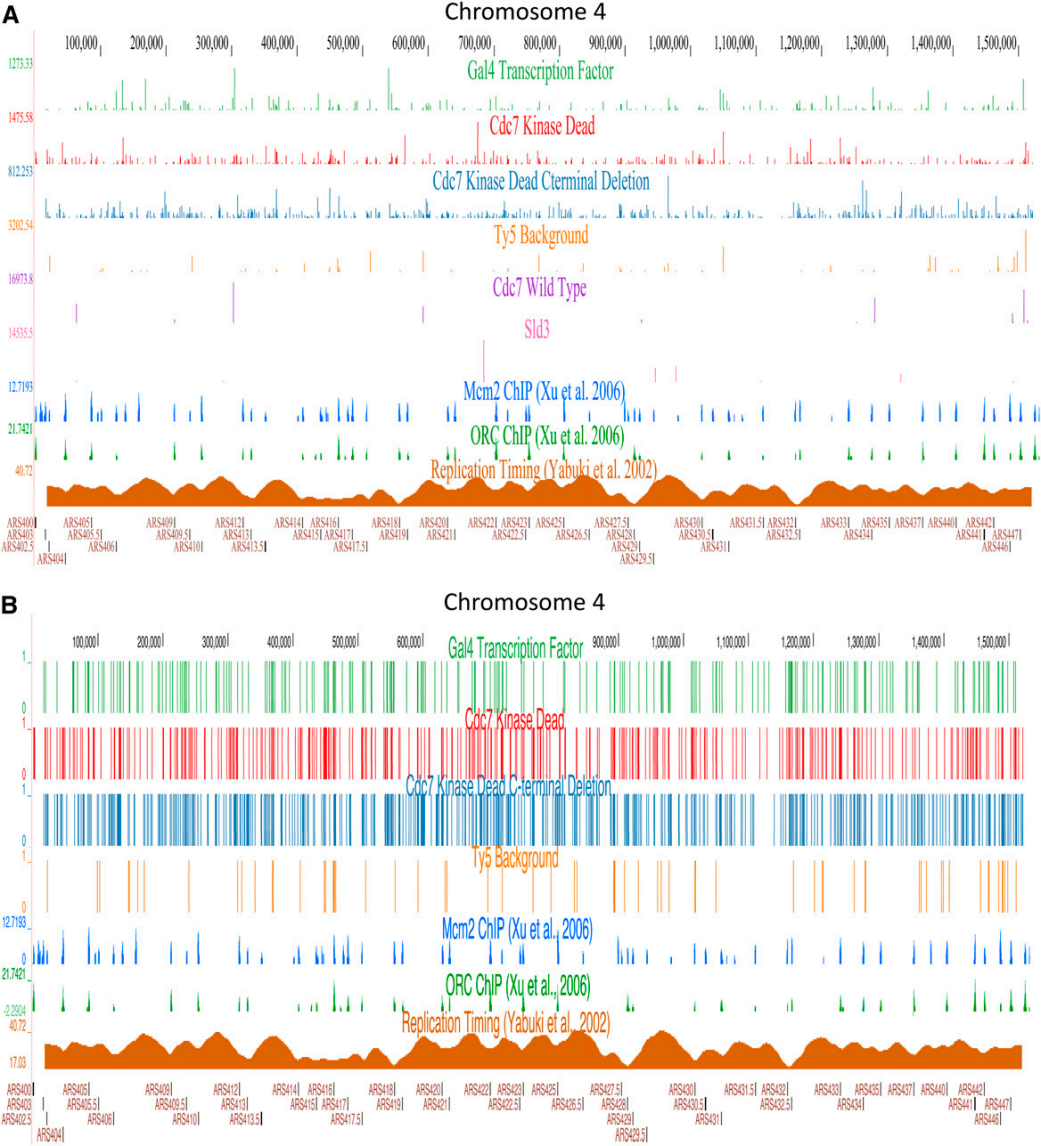
Calling Cards sequences mapped to *S. cerevisiae* genome. Ty5 transposons integrated by DNA replication factor fusion constructs were isolated, sequenced, and mapped to the yeast genome. Chromosome 4 from the UCSC genome browser is used to show where Ty5 transposon integration marks occur for each DNA replication factor fusion construct. Orc1 and Mcm2 chromatin immunoprecipitation (Xu *et al.* 2006) and replication timing (Yabuki *et al.* 2002) are plotted for reference. (A) Calling Cards signal at each position normalized to the number of aligned reads in the library (reads per million, RPM). The *X*-axis and vertical lines represent Ty5 transposon integration events at specific positions within the chromosome and the *y*-axis and height of each line is the normalized signal at each position. (B) Normalized to mean number of unique insertions events. Removes quantitative information and only displays unique position of insertion events in the genome.

Upon high-throughput sequencing of genomic DNA flanking Ty5 integrations, the number of integration events retrieved for each construct was determined (*cdc7KD-sir4* ∼5000 insertions, *cdc7KDΔCT-sir4* ∼9000 insertions, *Gal4-Sir4* ∼5600 insertions and Ty5 background ∼2100 insertions). *cdc7KD-sir4* and *cdc7KDΔCT-sir4* fusions resulted in more Ty5 transposon integrations throughout the genome compared to the *CDC7-sir4* and *SLD3-sir4* constructs and to the strain containing only the Ty5 donor plasmid as expected due to their low integration inefficiencies. We show chromosome 4 from the UCSC genome browser as a representation for where each fusion construct integrated Ty5 retrotransposons (Figure 2). Each insertion event represents a location where Gal4 or Cdc7 proteins were associated with chromatin. We normalized the Calling Cards signal using two different methods. Initial Calling Cards analysis was done by taking the number of reads at each genomic position (the signal) and normalizing the signal to the total number of aligned reads in the library for a given construct which provides output as RPM. Here, each individual peak represents an insertion event at a specific location, while the height of each peak at a given location is the signal amplitude or the number of reads of that sequence due to either multiple insertion events at the same location or inverse PCR amplification bias of a single insertion event. As the *GAL4-sir4* fusion construct was used as a template to create the other construct, the backbone of the constructs were not changed to include unique molecular identifiers that would have allowed separation between unique insertion events and insertion events that had inherent amplification bias in our PCR step. The second analysis normalized Calling Cards to the mean number of unique insertion events, which ignores quantitative information and only provides information as to where insertions occur. If an integration event occurs, the “count” is 1; if no integrations occur, the “count” is 0. Here, each peak is the same height, and we only look at unique positions where insertions occur.

When normalized to the number of aligned reads (RPM) (Figure 2A), *GAL4-sir4* fusion, as a positive control for Calling Cards, targeted Ty5 transposon integration throughout the genome as well as at all of its known major binding sites. *GAL4-sir4* inserted multiple Ty5 transposons at the promoters of each galactose metabolic gene including *GAL1*, *GAL10*, *GAL7*, *GAL2*, and *GAL3* as expected (Figure S1 in File S1). In addition to the galactose metabolic genes, *GAL4-sir4* integrated Ty5 transposons at other known *GAL4* binding regions including the promoters of *FUR4*, *GCY1*, and *PCL10* genes, among others (data not shown). The negative control strain containing the Ty5 donor plasmid without a Sir4 fusion construct reduced the signal of integrated Ty5 transposons in the genome dramatically compared to the *GAL4-sir4* fusion. Due to their low integration efficiency, *CDC7-sir4* and *SLD3-sir4* fusions integrated very few Ty5 transposons into the genome. We predict there is an integration efficiency threshold required for genomic coverage of potential Ty5 transposon integration events and these two constructs do not achieve the necessary threshold. Due to their high integration efficiency, *cdc7KD-sir4* and *cdc7KDΔCT-sir4* fusions are above the threshold and provide enough insertion events to significantly cover the genome. The two kinase dead constructs integrated Ty5 transposons throughout the entire budding yeast genome, similar to the *GAL4-sir4* fusion. To see the RPM signal of Ty5 transposon integrations across all 16 chromosomes, please use the following link to the interactive UCSC genome browser: http://genome.ucsc.edu/cgi-bin/hgTracks?hgS_doOtherUser=submit&hgS_otherUserName=drossbach&hgS_otherUserSessionName=Cdc7%20Calling%20Card%20Paper.

Upon normalization to the mean number of unique insertions events (Figure 2B), Ty5 transposon integrations are still made throughout entire chromosomes by the *GAL4-sir4*, *cdc7KD-sir4*, and *cdc7KDΔCT-sir4* fusions. To see the mean number of unique insertions across all 16 chromosomes please use the following link to the interactive UCSC genome browser: http://genome.ucsc.edu/cgi-bin/hgTracks?hgS_doOtherUser=submit&hgS_otherUserName=drossbach&hgS_otherUserSessionName=Calling%20Card%20Not%20%23%20reads%20paper. Using both normalization techniques, Ty5 transposon integrations are found throughout the genome and not specifically at origins of replication. Cdc7 binding to chromatin at nonorigin DNA may be indicative of its role in other cellular processes such as chromatid cohesion, TLS, and meiotic recombination.

As Cdc7’s main role is in DNA replication, we expect Ty5 transposon integrations to occur specifically at origins of replication as a result of Cdc7 binding to origins. Specific origins of replication were chosen in the UCSC genome browser to determine if Ty5 transposon integrations are made in relation to origins (Figure S3 in File S1). This analysis showed some origins contain Ty5 transposon integrations nearby (ARS1, ARS305), while other origins do not contain integrations or contain integrations >1 kb away (ARS306, ARS1010, ARS607) from the origin, which likely indicates a different Cdc7 binding site than the origin itself. Additionally, some Ty5 transposon integrations are made in genomic DNA that are known to bind the MCM complex, but do not bind the ORC complex (ARS308). Using the UCSC genome browser, there does appear to be a pattern as to which origins are susceptible to Ty5 transposon integration events.

As the UCSC genome browser is only useful for looking at chromosome-wide Ty5 transposon integrations, meta-analysis was performed to make further comparisons and determine if a difference exists in Ty5 transposon integration events from *cdc7KD-sir4* and *cdc7KDΔCT-sir4*. Our meta-analysis focused on whether Cdc7-directed Ty5 transposon integrations are dependent on Dbf4 binding, if Ty5 integrations correlate to chromosomal replication features including timing, ARS sites, and replication proteins, and whether correlations exist with nonreplication chromosomal features including transcription start sites and centromeres.

### Cdc7 directs Ty5 transposon insertions into early replicating DNA

To further understand the binding differences of kinase dead Cdc7 constructs to chromatin, Ty5 transposon integration events were compared to known DNA replication features. Calling Cards data were first organized by timing of genomic DNA replication across entire chromosomes as defined by previous microarray data sets (Raghuraman *et al.* 2001). Newer sequence-based replication timing data (Muller *et al.* 2014) was looked at for comparison and as no significant differences existed from microarray data sets, Calling Cards data were not reanalyzed with these data. The replication timing data set was divided into 5-min windows by *T_rep_* and Calling Cards signal in each window was determined.

**Figure 3:**
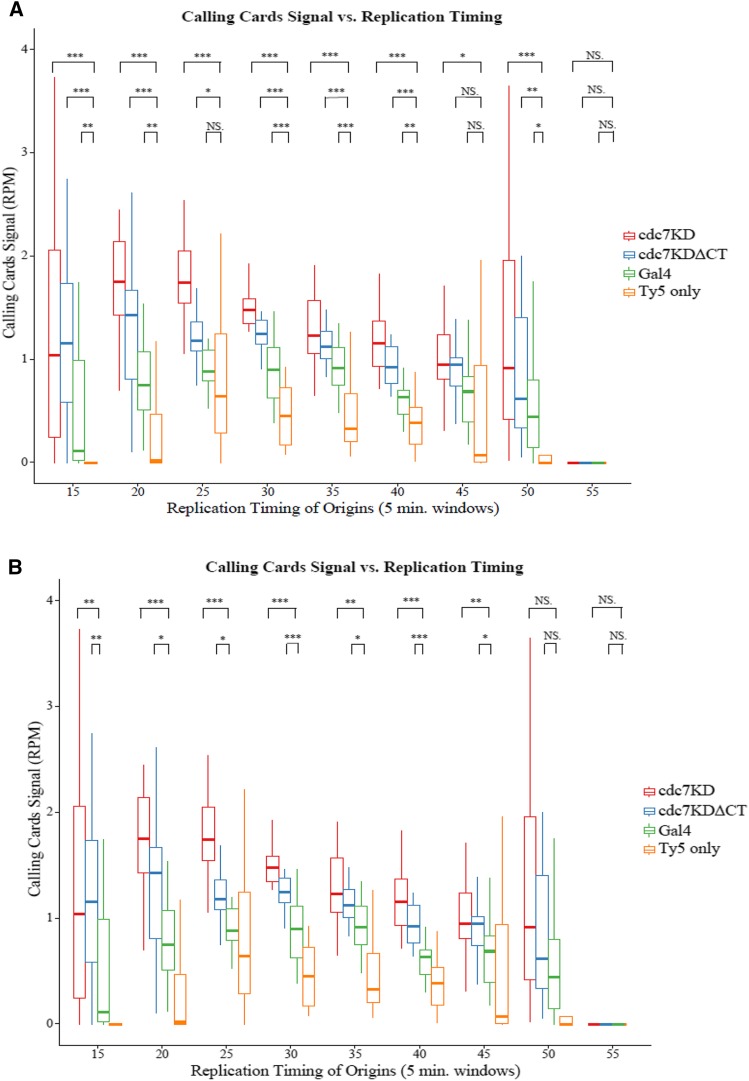

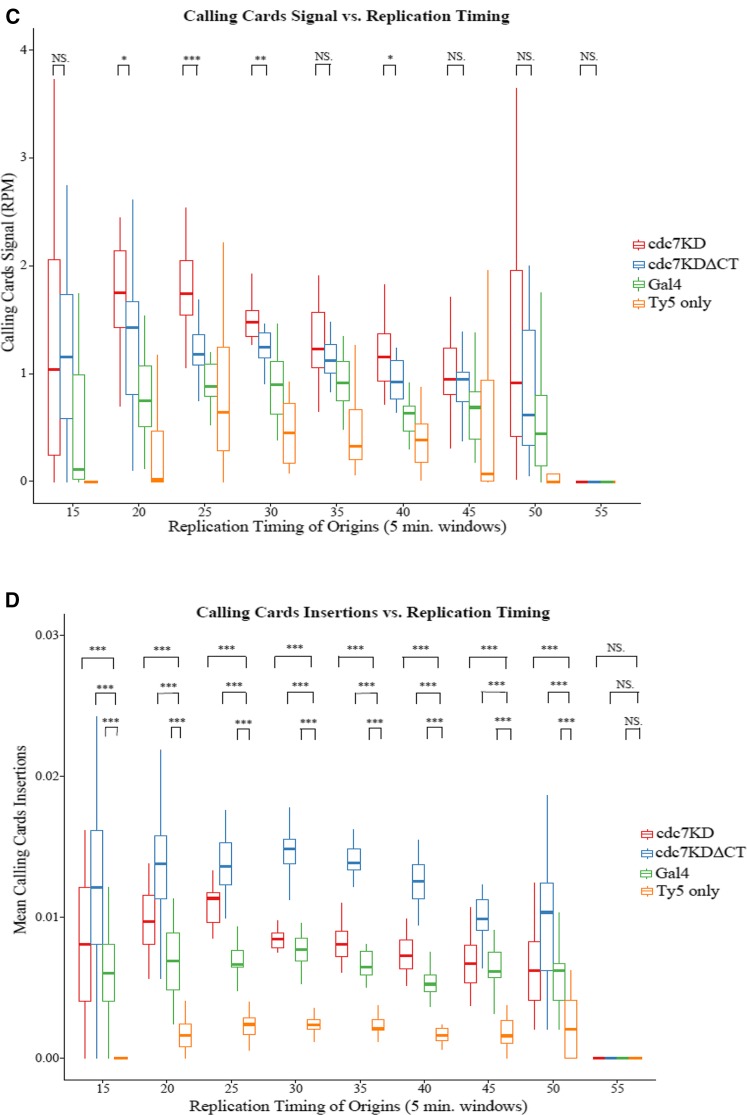

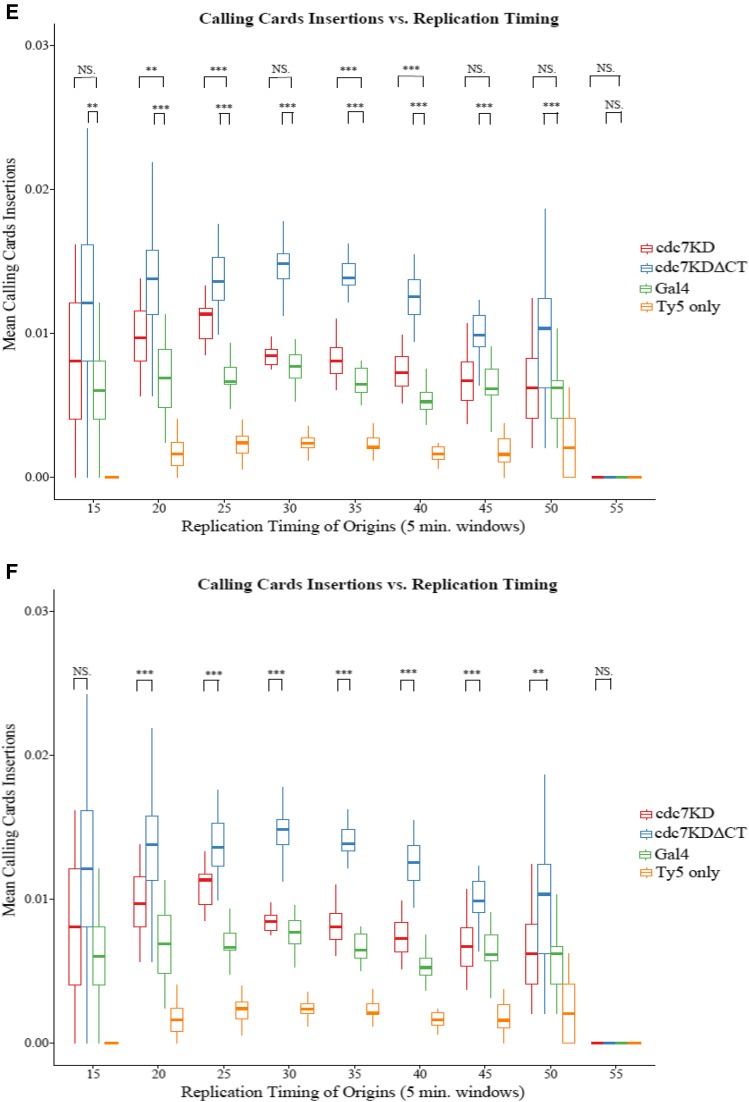
Ty5 transposons from Cdc7 insert into early replicating DNA. Ty5 transposon integrations are compared to timing of replication of the genome. Replication timing microarray data sets were used (Raghuraman *et al.* 2001). The genome was divided into 5-min windows based on the overall timing of replication. Each box is a representation of the Calling Cards signal of a given construct in a specific replication timing window. Calling Cards signal at each position was normalized to the number of aligned reads in the library (reads per million, RPM). The *X*-axis and vertical lines represent Ty5 transposon integration events at specific positions within the chromosome and the *y*-axis and height of each line is the normalized signal at each position. (A) RPM signal comparing each fusion construct to negative control Ty5 integrations. (B) RPM signal comparing Cdc7 fusion constructs to Gal4 fusion construct. (C) RPM signal comparing *cdc7KD-sir4* fusion construct to *cdc7KDΔCT-sir4* fusion construct. Calling Cards was normalized to mean number of unique insertions events. (D) Mean number of unique insertions comparing each fusion construct to negative control Ty5 integrations. (E) Mean number of unique integrations comparing Cdc7 fusion constructs to Gal4 fusion construct. (F) Mean number of unique integrations comparing *cdc7KD-sir4* fusion construct to *cdc7KDΔCT-sir4* fusion construct. Statistical analysis was done using the nonparametric Wilcoxon test as we were not convinced our data followed a normal distribution. *P = 0.01–0.05; ** *P* = 0.001–0.01; *** *P* < 0.001; NS, not significant.

When normalized to the number of aligned reads (RPM) (Figure 3, A–C), the negative control containing the Ty5 donor plasmid had low Ty5 transposon integration signal across each 5-min replication timing window as expected due to its low integration efficiency. Ty5 transposon integration signal due to the *GAL4-sir4* positive control was higher than the negative control and highest in regions of genomic DNA that are known to replicate in mid-S phase and low in regions of genomic DNA that replicate early or late in S phase suggesting integration events due to Gal4 binding do not correlate well with replication timing. Ty5 transposon integration signal due to *cdc7KD-sir4* fusion was higher than background and highest in regions of DNA that are the earliest replicating. The signal steadily decreased in regions that replicate late in S phase. Previous experiments showed the majority of yeast origins initiate replication within the first 20–40 min of S phase and as S phase progresses fewer origins are needed to initiate replication (Yang *et al.* 2010). Thus, in the same timing windows when the majority of origins initiate replication, the most Ty5 integrations were made due to Cdc7 binding chromatin. *cdc7KDΔCT-sir4* fusion had a similar trend to *cdc7KD-sir4* where Calling Cards signal peaked in regions of early replicating genomic DNA and was low in late replicating DNA. The Ty5 transposon integration signal from *cdc7KDΔCT-sir4* fusion was significantly lower than that of the *cdc7KD-sir4* fusion in the 15- to 30-min range. Removing the C-terminal of Cdc7, a known essential domain for Dbf4 binding, may have a negative effect on the amount of Cdc7 bound to chromatin suggesting a role for Dbf4 in stabilizing Cdc7-chromatin binding.

When Calling Cards data were normalized to the mean number of unique insertion events, providing only positional information (Figure 3, D–F), similar trends were seen in comparison to replication timing. *cdc7KD-sir4* and *cdc7KDΔCT-sir4* created unique Ty5 integrations into early replicating regions of the genome and *GAL4-sir4* integrated Ty5 primarily into mid-S regions, while the negative control integrations were low throughout all timing windows. However, with this normalization, *cdc7KDΔCT-sir4* produced a higher mean number of unique insertions than *cdc7KD-sir4*, a reversal of the RPM signal normalization. This suggests *cdc7KDΔCT-sir4* made more unique insertions throughout each timing window, but *cdc7KD-sir4* produced an overall higher average signal at specific locations.

### Comparison of Cdc7 to ACS sites

As it is difficult and time consuming to use the UCSC genome browser to assess patterns at all origins of replication, meta-analysis was performed to analyze patterns of Ty5 integration near all confirmed, likely, or dubious ARSs. These ARS classifications are determined by ACS sites in the genome and whether they are known to initiate replication. Confirmed or likely sites are ACS sites that are known to initiate replication while dubious sites are ACS sites that are not explicitly known to initiate replication. The list of these sites was taken from the *S. cerevisiae* origin database (http://cerevisiae.oridb.org/data_output.php?main=sc_ori&table=sc_ori&ext_format=BED;FASTA;&format=tab). For this analysis, we took the subset of integration events that occurred within the first 30 min according to the timing data and took a window containing 1-kb genomic DNA on either side of confirmed/likely ACS sites or dubious ACS sites to determine how Cdc7-chromatin binding correlates with ARSs. This 2-kb window around ACS sites was divided into 100-bp windows and the mean RPM or mean number of unique insertion events in each window was determined for the constructs.

**Figure 4:**
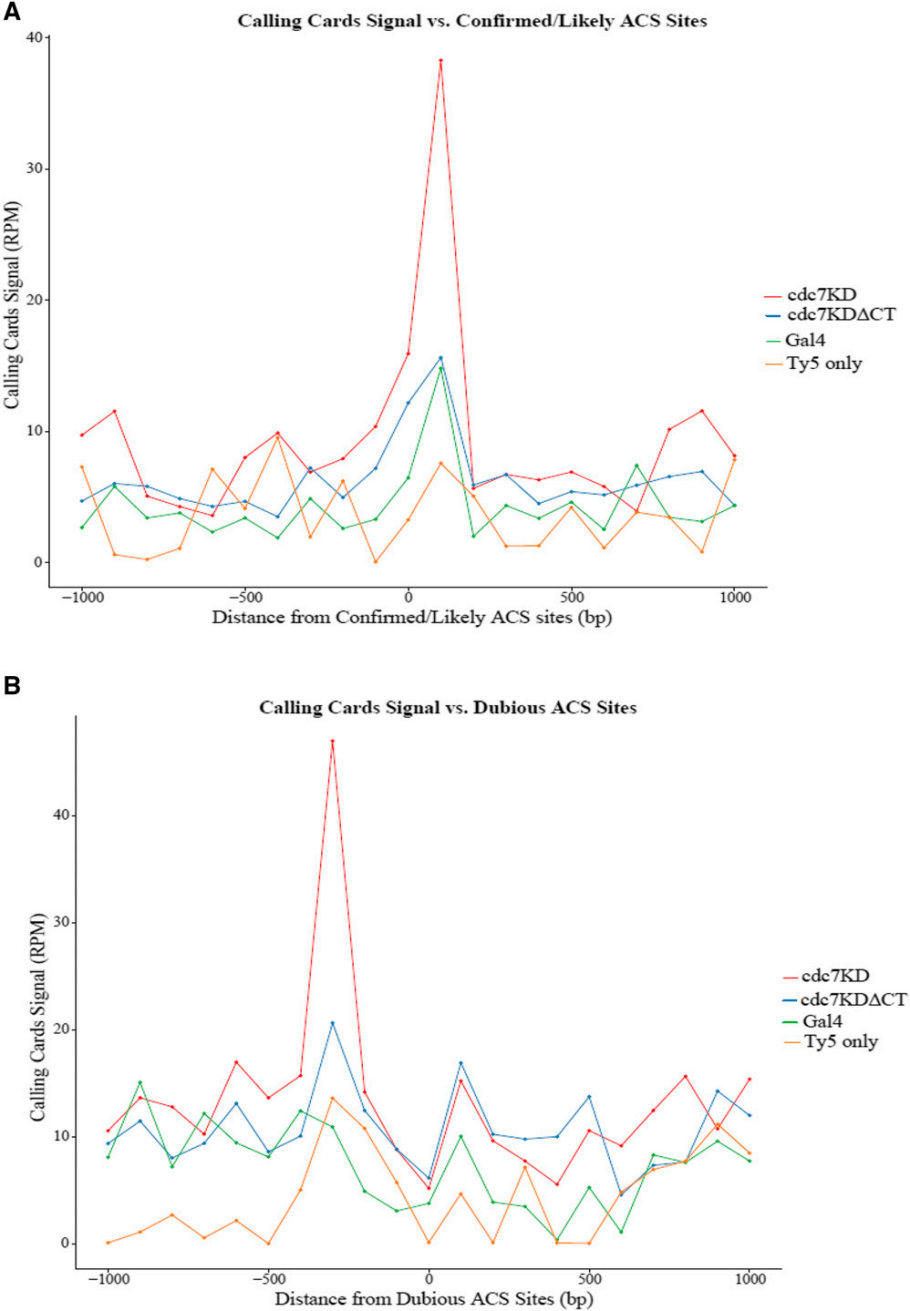

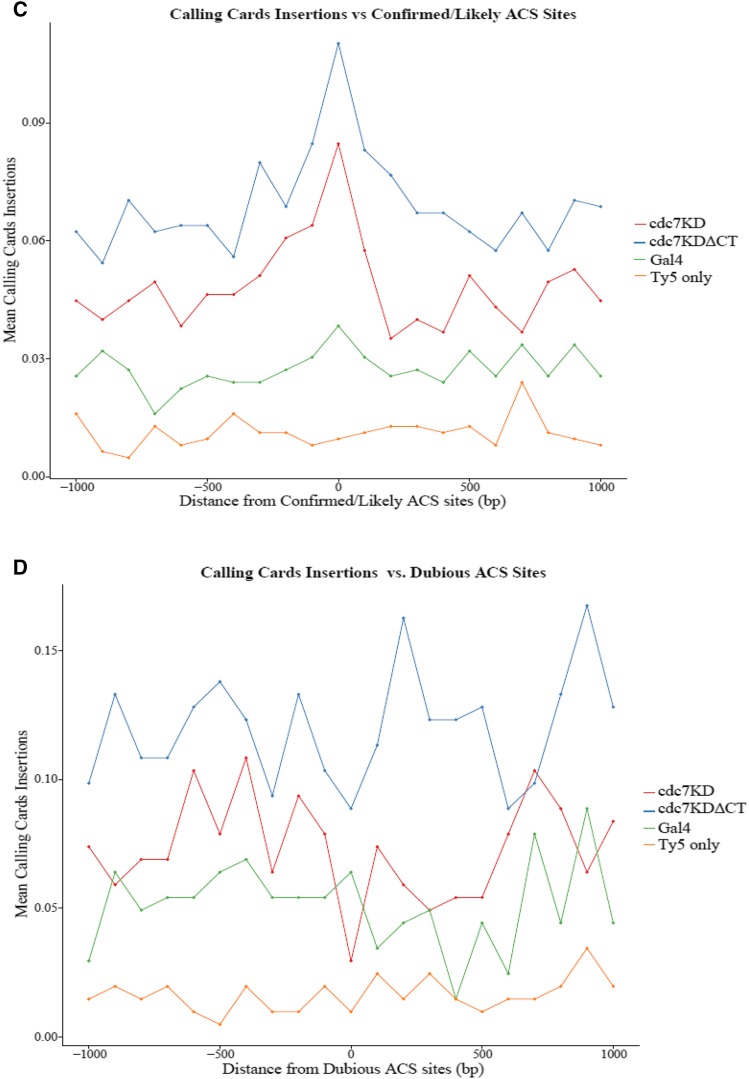
Ty5 insertions integrate preferentially near ACS sites. Ty5 transposon insertions are compared to known ACS sites within the genome. A 1-kb window on either side of ACS sites was used to determine where Cdc7-directed integrations occur in relation to the ACS. (A) Calling Cards insertions signal compared to confirmed/likely ACS sites normalized to the number of aligned reads in the library (reads per million, RPM). The *x*-axis is the position relative to ACS sites and *y*-axis is the normalized signal. (B) Calling Cards insertions signal compared to dubious ACS sites normalized to the number of aligned reads in the library (RPM). (C) Calling Cards insertions compared to confirmed/likely ACS sites normalized to the mean number of unique insertions where the *x*-axis is the position relative to ACS sites and *y*-axis is the mean number of insertions for a given genomic position. (D) Calling Cards insertions compared to dubious ACS sites normalized to mean number of unique insertions.

When Calling Cards data were normalized to the number of aligned reads (RPM) and comparing to confirmed/likely ACSs, *cdc7KD-sir4* fusion construct produced a sharp increase in the Ty5 transposon integration signal within 100 bp of the center of the ACSs relative to the surrounding genome (Figure 4A). This suggests Cdc7 binds preferentially near ACS sites known to initiate replication compared to surrounding genomic DNA. While Cdc7 does bind preferentially near confirmed/likely ACS sites, there is still Ty5 integration signal when we move away from the ACS sites, evidence that Cdc7 is bound and needed at other places in the genome beyond just origins. *cdc7KDΔCT-sir4* reduced the Ty5 integration signal at ACS sites by threefold suggesting a possible role for Dbf4 in stabilizing Cdc7 on chromatin or at least at specific sites. At dubious ACS sites, *cdc7KD-sir4* produced a peak in Ty5 transposon integration signal that was shifted to 300 bp away from the center of the proposed ACS site compared to confirmed/likely ACS sites. As Cdc7 is still bound near dubious ACS sites, it is possible Cdc7 still recognizes the sequence specificity of an ACS site as positions where it should bind even if that site doesn’t become a functional origin (Figure 4B). Similar to our ARS308 which binds MCM complex and not ORC, these dubious sites may still bind MCM to promote Cdc7 association even when ORC is not bound. This sharp increase of Cdc7 binding specifically near ACS sites is consistent with the idea that Cdc7 is bound to specific sequences that will become origins of replication to promote replication initiation.

When Calling Cards data were normalized to the mean number of unique insertion events, *cdc7KD-sir4* again produced a peak in the number of unique integrations near confirmed/likely ACS sites (Figure 4C). As with replication timing, *cdc7KDΔCT-sir4* made more unique integration events near confirmed/likely ACS sites than *cdc7KD-sir4*. It is not known why the unique insertion events near dubious ACS sites is much more variable than that at confirmed/likely ACS sites, such that the mean number of integration events decreased immediately at the ACS site and contained no large peak within 300 bp as seen previously (Figure 4D). Comparing Cdc7-chromatin binding to known ACS sites as a marker for origins of replication indicates Cdc7 has a preference for binding sequences that could become origins.

### Comparison of Cdc7 to Orc1 binding sites

Calling Cards data were also compared to reported genomic binding sites of the replication protein Orc1 (Aparicio *et al.* 1997; Xu *et al.* 2006; Ramey and Sclafani 2014). Again, integrations that occurred within the first 30 min were used and analyzed in relation to 5-kb genomic DNA on either side of known Orc1 binding sites to determine how close to specific replication protein sites Cdc7 binds. This 10-kb genomic DNA around Orc1 sites was again broken into 100-bp windows where mean RPM and mean number of insertions was calculated.

**Figure 5:**
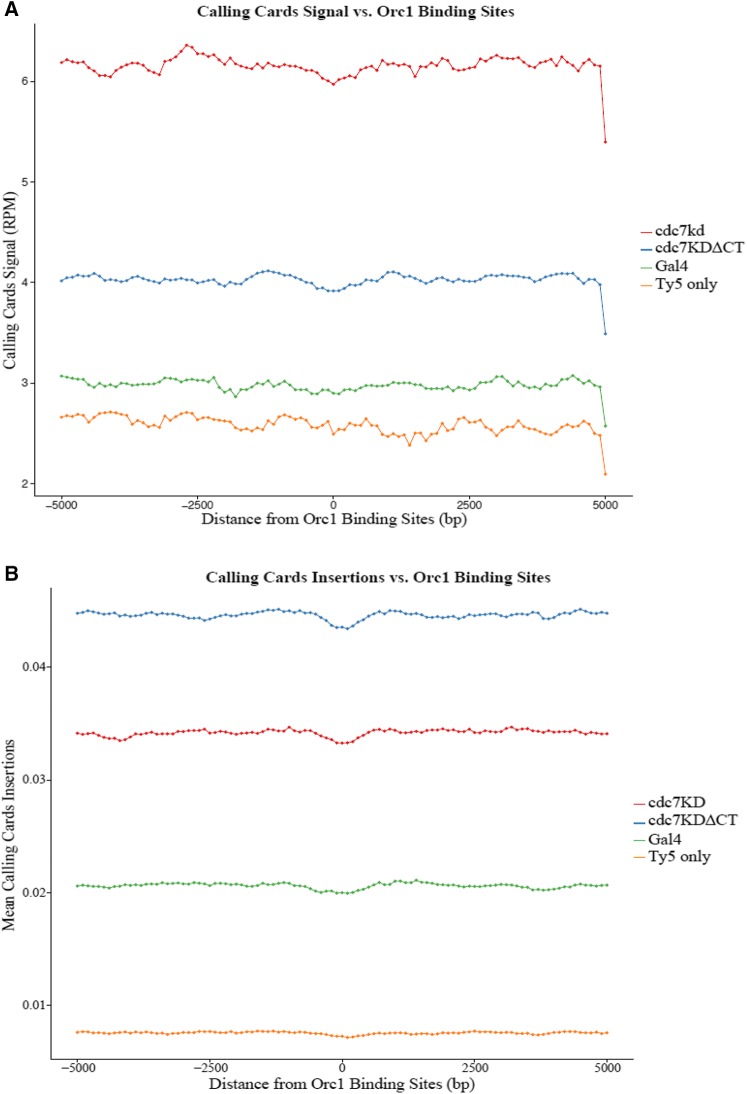
Ty5 transposons do not correlate with Orc1 binding sites. Ty5 transposon insertions compared to Orc1 binding sites. A 5-kb window on either side of all Orc1 binding sites was used to determine where integrations occur in relation to protein markers of replication. (A) Calling Cards insertions compared to Orc1 binding sites normalized to the number of aligned reads in the library (reads per million, RPM). The *x*-axis is the position relative to Orc1 binding sites and the *y*-axis is the normalized signal. (B) Calling Cards insertions compared to Orc1 binding sites normalized to mean number of unique insertions at a given position.

Gal4 has no specific role in DNA replication, and, as expected, *GAL4-sir4* mean RPM signal shows little variability in intensity across each 100-bp window in the entire 10-kb genomic segments. Additionally, the Ty5 integration signal due to *GAL4-sir4* was not increased substantially compared to the negative control containing only the Ty5 donor plasmid. Gal4 has no preference to bind near Orc1 binding sites. When normalized to the number of aligned reads (RPM), *cdc7KD-sir4* and *cdc7KDΔCT-sir4* had little variability in Ty5 transposon integration signal intensity across the genomic segment but rather appear to direct Ty5 transposon integrations across the entire 10-kb window to the same levels like Gal4 (Figure 5A). The signal intensity of *cdc7KD-sir4* integrations was increased more than twofold compared to the negative control and *GAL4-sir4*. *cdc7KDΔCT-sir4* Ty5 transposon integration signal was reduced in the region around Orc1 by an order of magnitude compared to *cdc7KD-sir4*, suggesting Dbf4 binding affects Cdc7 binding to chromatin. While Cdc7 constructs increased the Ty5 transposon integration signal, the integrations still did not show a preference for inserting specifically near Orc1 binding sites as may be expected since Orc1 is bound to all functional origins.

When normalized to the mean number of insertion events, all constructs had little variability in Ty5 transposon integration signal near Orc1 binding sites (Figure 5B). As with the other meta-analysis, *cdc7KDΔCT-sir4* again showed a higher number of unique insertions around Orc1 binding sites relative to *cdc7KD-sir4*.

Cdc7-directed insertions do not bind preferentially to Orc1 binding sites, indicating there are likely more binding sites for Orc1 than only at the ACS sites. As a protein, it is possible there are more possibilities for where Orc1 can bind and thus Cdc7 insertions may be distributed more evenly in relation to these sites. In addition to specific DNA replication features, Calling Cards data were compared to other chromosomal features including transcription start sites (TSS) and centromeres (Figure S3 in File S1). Cdc7 binding does not correlate with TSS but does bind in pericentric genomic DNA, suggesting it is correlated with centromeric DNA to carry our chromatid cohesion.

### Cdc7 is bound to early origins as cells are released from S phase

**Figure 6:**
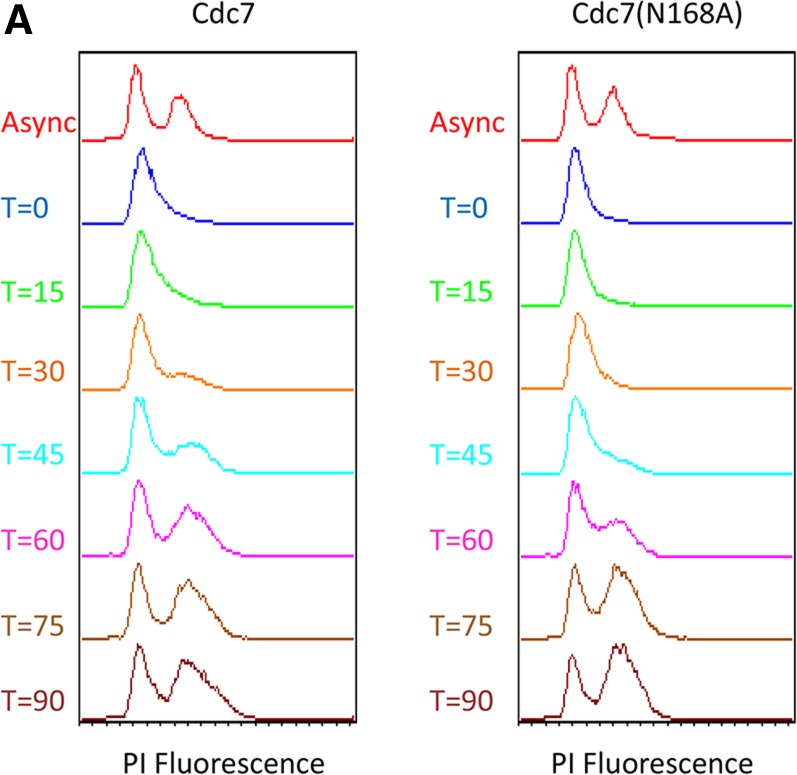

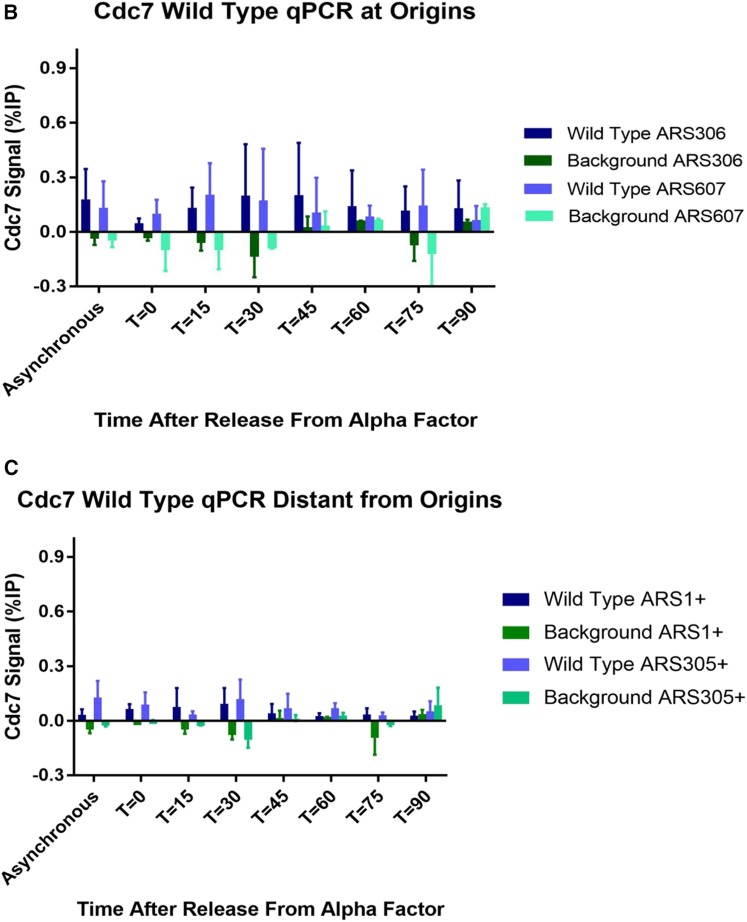

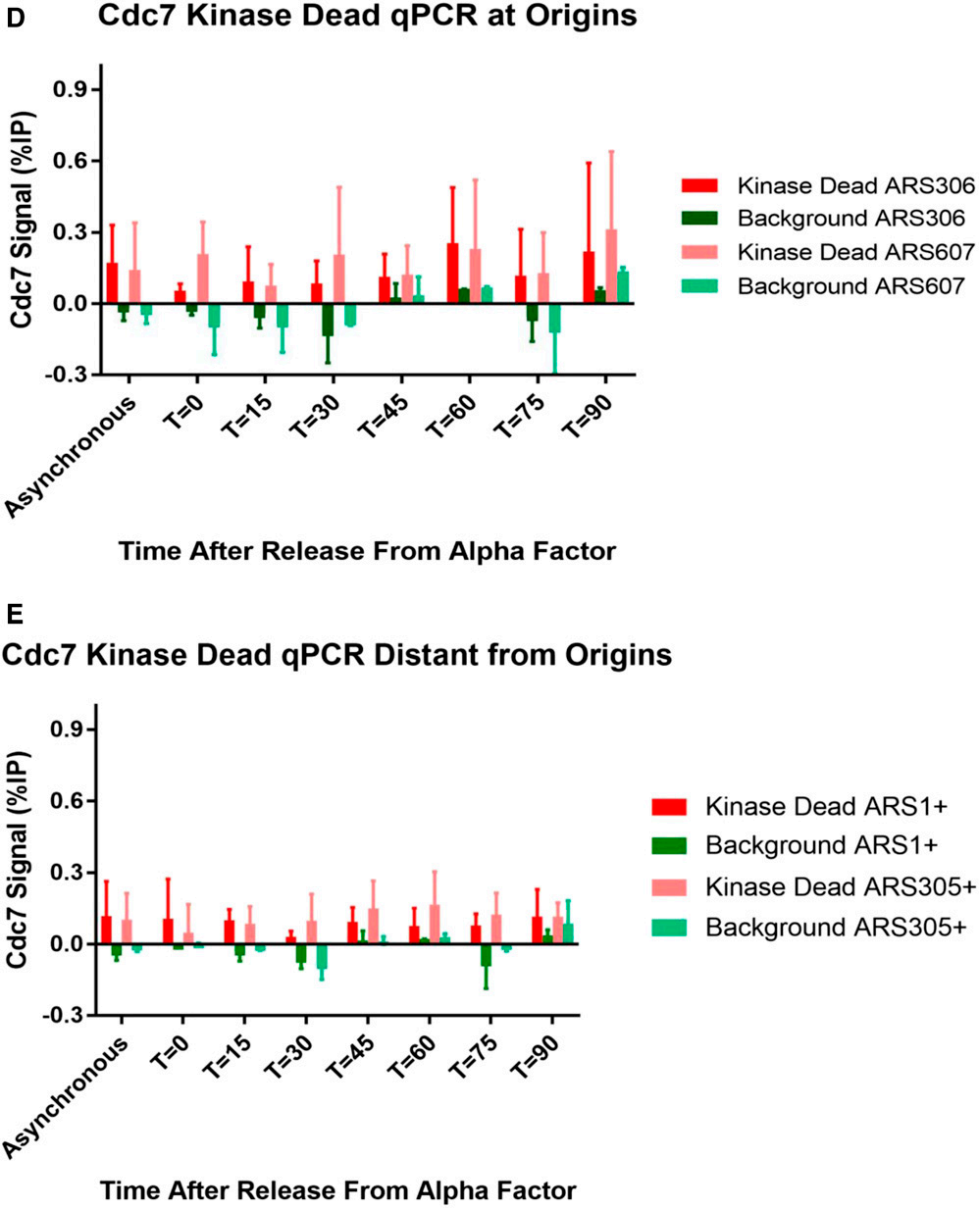
Chromatin immunoprecipitation (ChIP) analysis of Cdc7 at origins of replication. ChIP in strains overexpressing wild-type or kinase dead Cdc7 and strains lacking overexpression was performed on cells released from G1 arrest through S phase using an anti-HA antibody or no antibody negative control. Chromatin associated DNA was analyzed using quantitative PCR for specific DNA segments. (A) Flow cytometry analysis of wild-type and kinase dead Cdc7 overexpression plasmids. (B) ChIP analysis of wild-type Cdc7 using primers specific to origins of replication in a strain with overexpression or no overexpression. (C) Similar ChIP analysis of wild-type Cdc7 using primers specific to genomic regions 10 kb distant from ARS1 and 8 kb distant from ARS305. (D) ChIP analysis of kinase dead Cdc7 using primers specific to origins of replication in strains with or without overexpression. (E) ChIP analysis of kinase dead Cdc7 using primers specific to genomic regions distant from origins.

ChIP using endogenous Cdc7 protein has been technically ineffective as reliable results have not been obtained, even when Dbf4 binding was detected (Dowell *et al.* 1994; Katou *et al.* 2006). To further confirm whether Cdc7 was bound to specific origins of replication, ChIP was used in yeast strains that contain overexpressed Cdc7 wild-type and cdc7KD [Cdc7KD(N168A)] mutant proteins. Plasmids containing HA-tagged wild-type *CDC7* or *cdc7KD* mutant were transformed into strain RSY1294 (Table 1) that maintains endogenous Cdc7 protein. Flow cytometry confirmed overexpression of either Cdc7 or cdc7KD does not affect progression through S phase of the cell cycle as seen previously with the cdc7(N168A) mutant (Hardy 1997). Cells containing either overexpression protein began to release from G_1_/S arrest between 30 and 45 min after addition of pronase and completed S phase at ∼90 min (Figure 6A).

Early origins were used to determine association of Cdc7 protein with specific origins at various points during S phase. Both ARS306 and ARS607 have timing of replication between 10 and 13 min (Raghuraman *et al.* 2001; Yabuki *et al.* 2002; Alvino *et al.* 2007). Wild-type Cdc7 protein at these origins increased at 30 min after release, coinciding with the release into S phase from the G_1_/S arrest (Figure 6B). Cdc7 protein signal at both origins peaked at ∼45 min parallel to the timing of replication of 10–13 min. Previous chromatin fractionation experiments showed an increase in Cdc7 protein bound to bulk chromatin as cells enter into S phase (Weinreich and Stillman 1999). We used a negative control without an HA-tagged protein to show that increases during specific time points were specific to HA-Cdc7 protein. The negative control did not show signal increases at origins during S phase.

Similar to wild-type Cdc7 protein, Cdc7KD(N168A) protein signal increased at 30 min after release from S phase. However, Cdc7KD(N168A) signal peaked at ∼60 min, 15–30 min later than what was observed for wild type potentially due to Cdc7KD(N168A) binding longer. This kinase dead Cdc7 protein is still able to bind but cannot phosphorylate the MCM complexes. Both Cdc7 and Cdc7KD(N168A) signal decreased briefly ∼75 min before increasing again at 90 min (Figure 6D).

Primers corresponding to DNA 10 kb away from ARS1 and 8 kb away from ARS305 were used to determine Cdc7 binding to regions of the genome not containing origins (Table 3). While wild-type Cdc7 could not be significantly detected at either of these nonorigin regions, Cdc7KD(N168A) was found at these regions specifically between 60 and 75 min (Figure 6, C and E), again likely reflecting Cdc7KD(N168A) interacting with the substrate longer and not exhibiting transient binding similar to wild-type Cdc7 protein.

## Discussion

Using modified Calling Cards and ChIP methods in *S. cerevisiae*, we have begun to map Cdc7 protein kinase binding to chromatin during DNA replication. Identifying Cdc7 binding patterns and potential specificity within the genome furthers our understanding of the underlying molecular mechanisms of DNA replication initiation. It has been established that Cdc7 activity is required throughout S phase to initiate replication from both early and late firing origins. Our Calling Cards results indicate the greatest amount of Cdc7 kinase is bound to early replicating regions of the genome when the majority of origins are used to initiate replication. As cells progress through S phase, fewer origins are activated and less Cdc7 kinase is needed and bound in regions of late replicating DNA.

Calling Cards analysis showed wild-type Cdc7 or other replication proteins inefficiently integrate Ty5 transposons into the genome, likely due to the fact that Cdc7 phosphorylation is a transient process as expected for an enzyme and the MCM helicases or other substrates are not bound long enough to allow for efficient Ty5 transposon integration. Kinase dead Cdc7 protein binds the MCM helicases long enough to allow a more efficient integration of the Ty5 transposon. Each of our kinase dead Cdc7 constructs binds to chromatin throughout the entire yeast genome as well as specifically at origins of replication. Furthermore, our results suggest that physical interaction between Cdc7 and the Dbf4 regulatory subunit protein may be required for Cdc7 binding specificity in the genome. When Cdc7 kinase dead protein lacking 55 C-terminal amino acids essential for binding Dbf4 is used, more unique insertions are made, but the overall Ty5 transposon integration signal is reduced. This suggests Cdc7 that cannot bind Dbf4 has a wider distribution on chromatin, and direct interaction between the two subunits confers more specificity as to where Cdc7 is binding especially at origins of replication.

Through Calling Cards meta-analysis, we determined Cdc7 does bind preferentially near known ACS sites, whether they are confirmed or still in doubt. Additionally, Cdc7 binding does not correlate with Orc1 binding sites indicating Cdc7 recognizes specific sequences in the genome in order to bind rather than recognizing bound protein complexes or substrates. Using ChIP, we found Cdc7 binding to specific early origins of replication. This association with early origins of replication changes throughout S phase such that Cdc7 is bound highest as cells release into S phase but decreases as cells complete S phase. The discrepancy between low wild-type Cdc7 binding in Calling Cards and the more substantial binding seen in ChIP may be attributed to the formaldehyde fixation step in the ChIP protocol. In ChIP, we fix the proteins to chromatin and stabilize the interaction, while Calling Cards maintain the transient nature of the kinase interaction.

Both Calling Cards and ChIP show Cdc7 overexpression is critical to effectively look at the relationship between Cdc7 and origins. Not only is Cdc7 bound to ACS sites and origins of replication, but Cdc7 is also bound to nonorigin DNA. We predict Cdc7 kinase activity is needed in other genomic DNA regions and not just origins to carry out functions in TLS and meiotic recombination in addition to replication and chromatid cohesion. Sequence specificity *vs.* substrate specificity is further characterized by correlation of Cdc7 binding with pericentric DNA. Centromeres are known to replicate early and our results provide evidence that Cdc7 is bound both in these early replicating regions as well as in pericentric DNA. We predict Cdc7 is bound in the centromeric flanking DNA to aid in chromatin cohesion.

**Figure 7:**
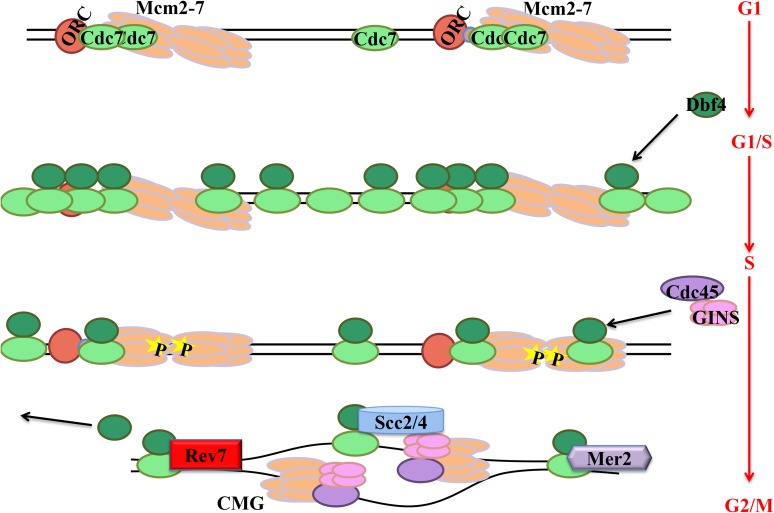
Model of Cdc7 action during DNA replication. Cdc7 protein is bound to chromatin throughout the genome during G1 phase of the cell cycle in the absence of Dbf4 protein. As Dbf4 protein expression increases, Cdc7 protein is stabilized on chromatin throughout the genome and is preferentially bound to known ACS sites. Increases in binding increase the potential to phosphorylate substrates including minichromosome maintenance (MCM) helicase complexes. Phosphorylation of the MCM complexes by Cdc7-Dbf4 kinase (DDK) results in loading of Cdc45 and GINS to form the active MCM helicase and to load on the replisome, which replicates the DNA. DDK not bound preferentially to ACS sites is used to promote TLS, meiotic recombination, and chromatid cohesion.

This study is consistent with the idea Cdc7 is bound specifically to origins to initiate DNA replication, but it also provides supports for a model in which Cdc7 can be bound throughout the genome even in G_1_ cells. Our model proposes that as cells enters the G_1_/S transition, Dbf4 protein expression increases, stabilizes Cdc7 on the chromatin, and allows preferential binding to origins. Precise localization of active Cdc7 at origins is important for DNA replication, but it is equally important to increase the total amount of Cdc7 bound to chromatin to initiate cellular processes beyond replication. This Cdc7 mass action increases the potential for phosphorylation of nearby MCM complexes or substrates for TLS, meiotic recombination, and chromatid cohesion (Figure 7) (Wan *et al.* 2008; Brandao *et al.* 2014; Princz *et al.* 2017). Our model is consistent with previous bulk chromatin studies in which Cdc7 was bound to chromatin throughout the cell cycle and increased after Dbf4 protein appeared in S phase (Weinreich and Stillman 1999). Recent ChIP experiments in G_1_-arrested cells used tagged Dbf4 proteins to show Dbf4 is bound weakly to origins (Katou *et al.* 2006; Natsume *et al.* 2013). However, levels of Dbf4 protein are very low in G_1_ (Cheng *et al.* 1999; Oshiro *et al.* 1999; Weinreich and Stillman 1999; Ferreira *et al.* 2000). In the previous studies, Cdc7 was not used, making the comparison difficult until ChIP could be modified. In our studies, Calling Cards provided evidence for preferential binding to ACS sites, and our modified ChIP experiments using overexpressed-tagged Cdc7 support this in which Cdc7 is closely associated with specific origins. Furthermore, our experiments are not restricted to G_1_-arrested cells and our analysis demonstrates how the Cdc7 interaction with origins changes at various time points in S phase. Together, our findings present a mechanism in which Cdc7 protein kinase binds throughout the genome using sequence-specific identification to carry out its many functions during the cell cycle and to promote DNA replication

## Supplementary Material

Supplemental material is available online at www.g3journal.org/lookup/suppl/doi:10.1534/g3.115.023366/-/DC1.
