# Supplementary material for: Localization of Cdc7 Protein Kinase During DNA Replication in Saccharomyces cerevisiae
Source: G3 (Bethesda). 2017 Sep 18;7(11):3757–74. doi: 10.1534/g3.117.300223 (PMC5677158; doi:10.1534/g3.117.300223)
Supplement: Supplementary file 1 [file 3757FileS1.pdf]

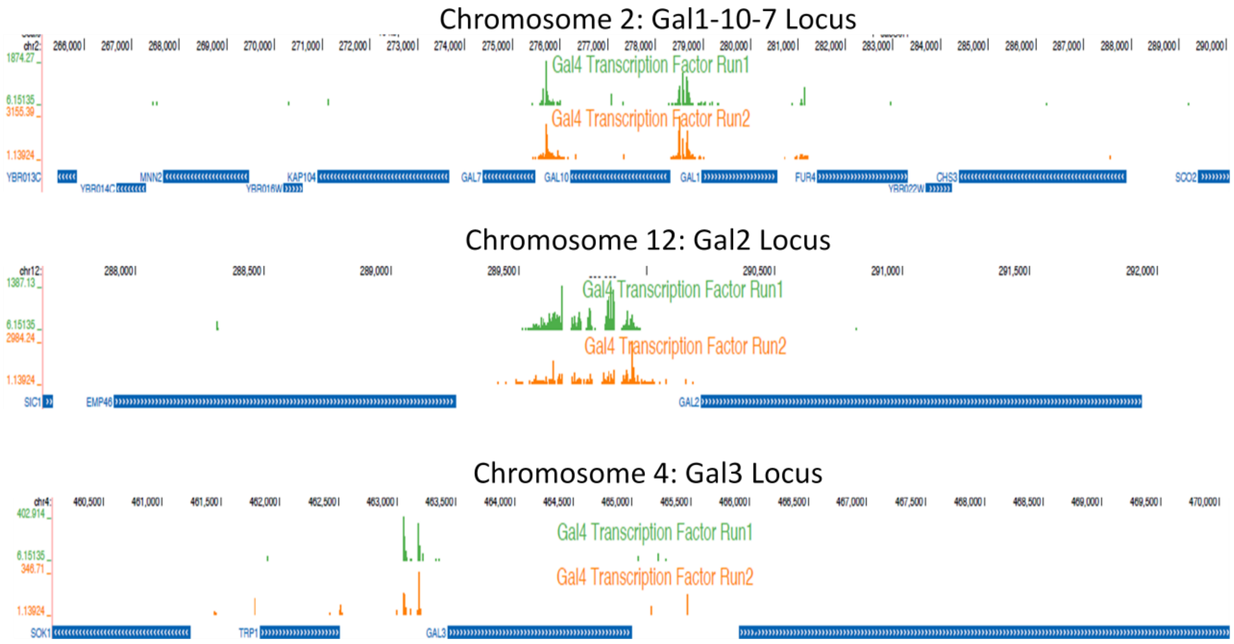

Figure S1. ***GAL4-sir4-myc* fusion construct integrates Ty5 at Galactose metabolic gene loci.** The GAL4-sir-myc fusion construct directs targeted integration of the Ty5 transposon into the genome near GAL transcription factor genes including the *GAL1-10-7* locus, *GAL2* locus, and *GAL3* locus. All contain strong Ty5 signals in the immediate vicinity of the genes.

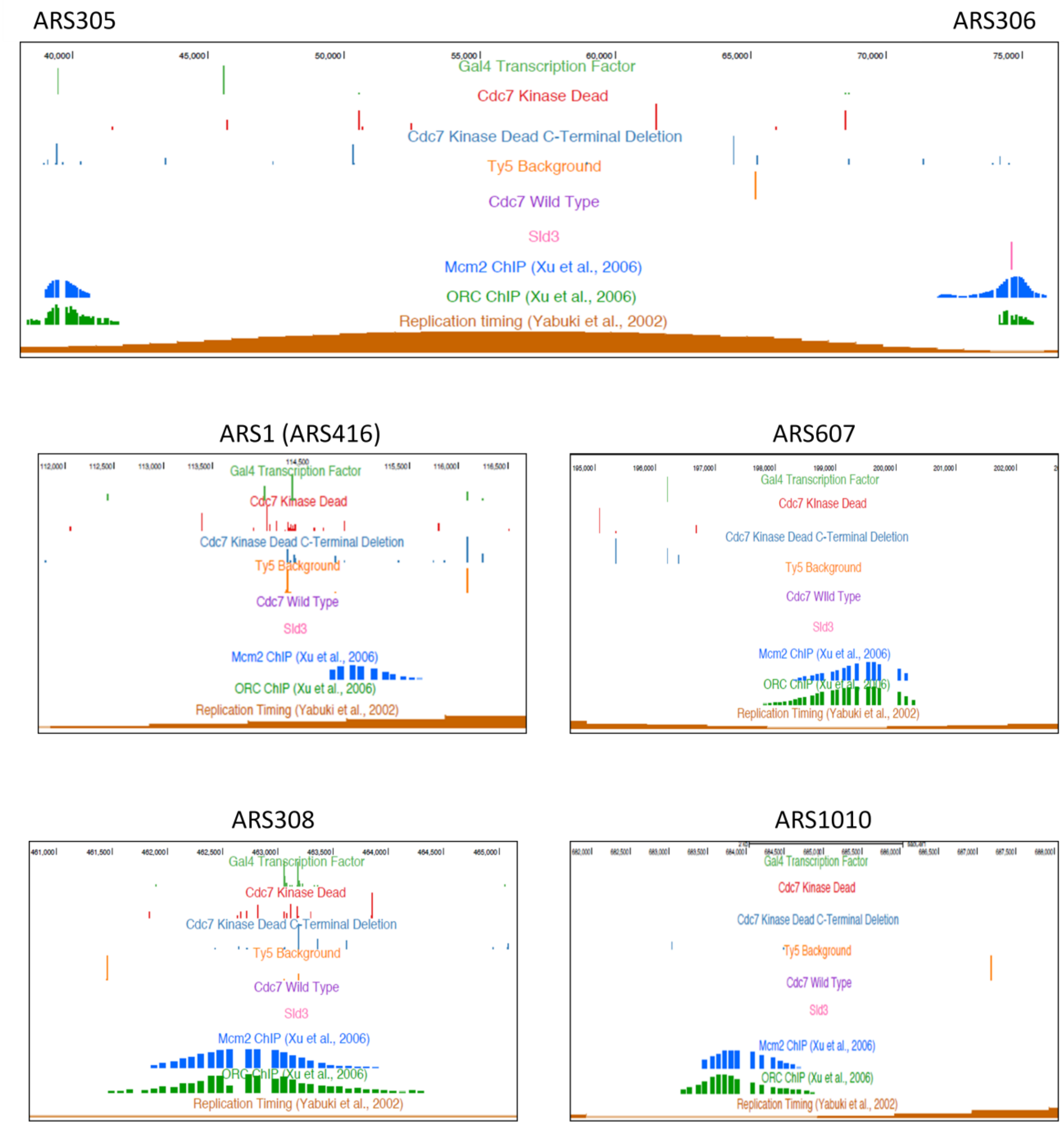

Figure S2. Ty5 Integrations at Origins of Replication.
Analysis of specific origins of replication shows varying Ty5 transposon integration signal. ARS305, ARS306, ARS1, and ARS607 were chosen to compare with ChIP results and ARS308 and ARS1010 were chosen at random. ARS305 and ARS306 contain few Ty5 integrations from *cdc7KDΔCT-sir4* and no integrations from *cdc7KD-sir4*. ARS1 contains robust Ty5 integrations from all constructs including *Gal4-sir4* transcription factor. ARS607 contains few Ty5 insertions from each construct that are 2-kb away from the origin. ARS308 contains many Ty5 insertions directly at the origin even though it is not bound by the ORC complex. ARS1010 is bound by ORC and MCM factors but does not contain any Ty5 insertions.

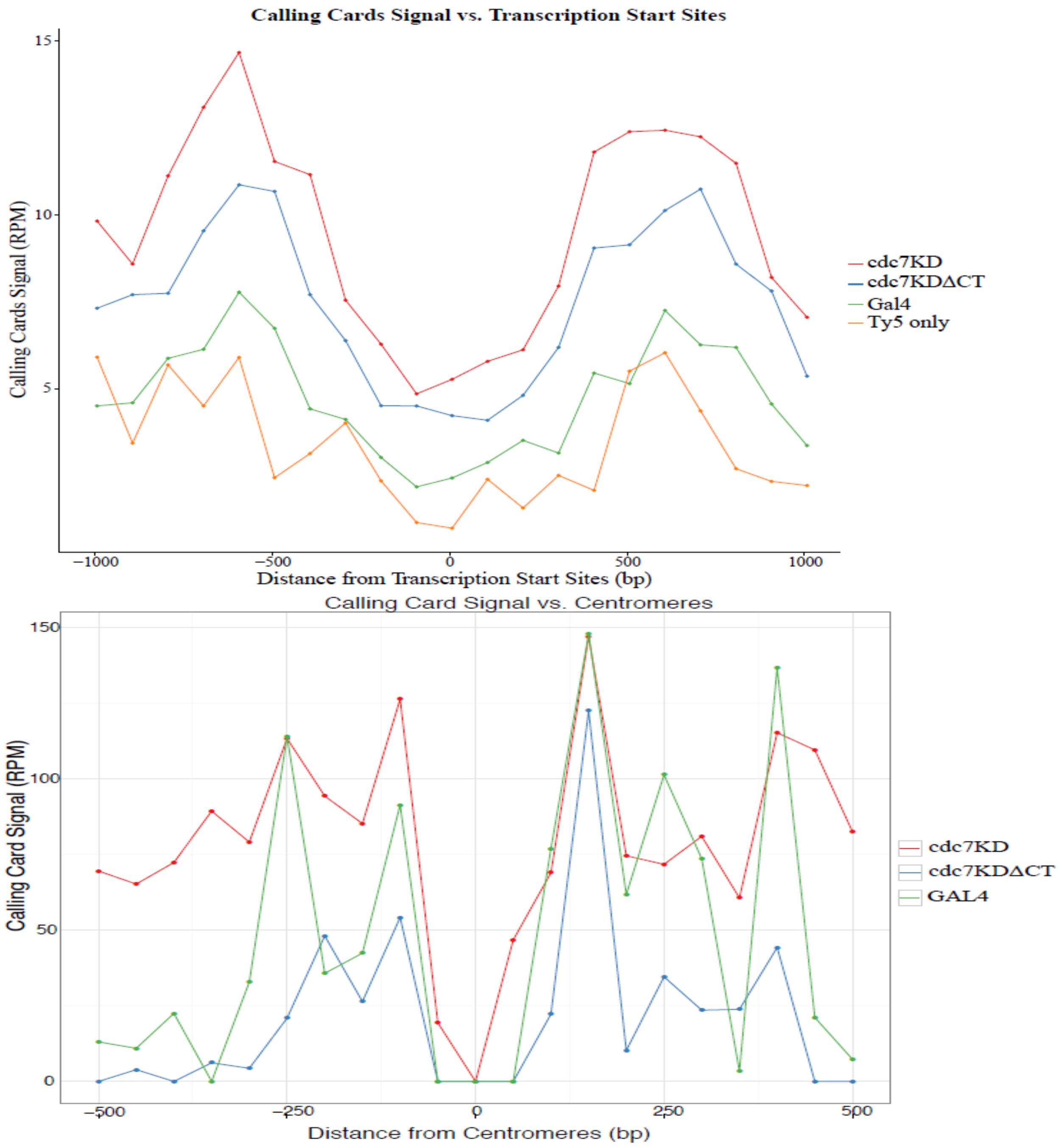

Figure S3. Ty5 Transposon Integrations, Transcription Start Sites and Centromeres. Ty5 transposon insertions compared to non-replication chromosomal features. Calling Cards compared to gene transcription start sites (TSS). 1-kb genomic DNA on either side of all (TSS) was used to determine where integrations occur in relation to transcription start sites. The X-axis is the position relative in the genome relative to TSS and the Y-axis is the relative number of reads aligned in the library (reads per million, RPM). Calling Cards insertions compared to centromeric DNA on chromosomes. 500-bp genomic DNA o either side of centromeres was used to determine where integrations occur relative to the centromere. The X-axis is the position on the chromosome relative to centromeres and the Y-axis is the relative number of reads aligned in the library (reads per million, RPM).
